# Modelling Interventions to Combat Antibacterial Resistance in East Africa Using Causal Bayesian Networks

**DOI:** 10.21203/rs.3.rs-5944839/v1

**Published:** 2025-02-04

**Authors:** Xuejia Ke, VA Smith, Stephen E. Mshana, Benon Asiimwe, Stella Neema, John Kiiru, Martha F. Mushi, Blandina T. Mmbaga, Joseph R. Mwanga, Gibson Kibiki, John Stelling, Stephen H. Gillespie, Dominique L. Green, Sepideh Benvari, Wilber Sabiiti, Mike Kesby, Andy G Lynch, Alison Sandeman, Derek J. Sloan, Matthew TG Holden, Katherine Keenan, HATUA Consortium

**Affiliations:** University of St Andrews; University of St Andrews; Catholic University of Health and Allied Sciences; Makerere University; Makerere University; Kenya Medical Research Institute; Catholic University of Health and Allied Sciences; Kilimanjaro Clinical Research Institute; Kilimanjaro Christian Medical Centre; Catholic University of Health and Allied Sciences; Africa Excellence Research Fund; Brigham and Women's Hospital; University of St Andrews; University of St Andrews; University of St Andrews; University of St Andrews; University of St Andrews; University of St Andrews; University of St Andrews; University of St Andrews; University of St Andrews; University of St Andrews; University of St Andrews

**Keywords:** antibacterial resistance, multidrug resistance, Bayesian networks, interventions, East Africa, systems modelling

## Abstract

Antibacterial resistance (ABR) poses significant challenges to combating infections worldwide. ABR drivers are interconnected, complicating identification of intervention points. Researchers need a systems-based perspective that considers interrelated drivers collectively. We focus on urinary tract infections (UTIs), which are increasingly impacted by emergence of multi-drug resistant (MDR) bacteria. We analysed 2,007 adult outpatients with UTIs in Kenya, Tanzania, and Uganda in 2019–2020. We applied structure learning in Bayesian networks, a graphical probabilistic model, alongside expert knowledge to construct a causal diagram of drivers of prevalence of MDR UTI. MDR prevalence was influenced more by demographic, socioeconomic and environmental conditions than recent antibiotic use. We conducted hypothetical interventions to estimate drivers’ causal effects, revealing that improving education access, providing protected drinking water and flush toilets, and reducing overcrowding would decrease MDR prevalence. A systems-based approach identified underlying causal patterns contributing to prevalence of MDR, and could guide the development of complexity-aware targeted interventions.

## Introduction

Bacterial antimicrobial resistance (ABR), particularly multi-drug resistance (MDR), is a major global health threat, leading to treatment failures and increased mortality and morbidity^1^. Urinary tract infections (UTIs) are common conditions that often require antibiotic treatment, and numerous studies have raised concern about rising MDR prevalence^2,3^. MDR in UTIs poses a particular threat in low-income settings like East Africa^4^, where high prevalence, combined with limited diagnostics and treatment options further increases clinical and societal burden.

### ABR Drivers and Intervention Points

The drivers of ABR/MDR are multifactorial and interact within a complex system^5,6^. UTI infections are predominantly caused by enteric Gram-negative bacteria colonizing the urethra and bladder^7^. In low- and middle-income countries (LMICs), human antibiotic (AB) use and ‘misuse’ (e.g., incomplete courses, using leftover antibiotics), and antibiotic use in livestock have been proposed to contribute to selection for and persistence of MDR in Gram-negative bacteria^8,9^. Public health factors like poor water, sanitation and hygiene (WASH) influence bacterial transmission, and thus burden of infections and antibiotic usage^10^. Factors contributing to both development of MDR and infection transmission include upstream living and working conditions which relate to broader socioeconomic and environmental vulnerabilities^11^. Most efforts to combat ABR in UTIs focus on targeted antibiotic use and stewardship, aiming to reduce selective pressure on pathogens, which would lower costs for treating recurrent and resistant UTIs^12^. However, in LMICs transmission factors may be as or more important to tackle, to reduce spread of already-MDR UTIs plus higher prevalence of UTIs increases pathogen population size and thus accelerates response to selection. Because factors influencing selection, transmission, and those upstream of both are interconnected, a systems-based approach is required to model how interventions operate within the wider network of ABR drivers.

### Causal Bayesian networks for modelling ABR interventions

Bayesian networks (BNs) offer a systems-based modelling approach that can handle complex, interconnected webs of variables. Invented by the statistician-philosopher Judea Pearl, BNs use directed acyclic graphs (DAGs) for structural representation and probabilistic models to estimate the dependencies between variables^13,14^. They are useful for understanding complex systems like ABR^15^, where traditional statistical methods often fail to distinguish between direct relationships and indirect or spurious associations. While applied widely in healthcare decision-making and gene expression analysis ^16–18^, their use for infectious disease is promising but still in its infancy^19–21^. Moving beyond interdependencies, causal BNs represent causal relationships that allow us to simulate interventions while taking account of confounding factors^22^. Following Pearl’s definition, a variable X is considered a cause of a variable Y if, on a population level, changes in X in any way decide the changes of values in Y^22^. Causal BNs can incorporate causal assumptions based on expert knowledge or empirical data, and have applications in health sciences^21,23^, engineering^24,25^ and machine learning (e.g., classification)^26^. These examples use associative reasoning in BNs, the first rung on Pearl’s “ladder of causation”^27^, where the probability of a target variable changes with the introduction of new evidence about other variables, serving as a decision-making tool^24^. Subsequent steps on the “ladder of causation” include a counterfactual approach, which we apply in this study^24^. This reflects a move from associative to predictive reasoning, which rather than describing how factors are related (associated), predicts how a population’s distribution would differ given modifying the population to fix particular factors. For example, while associative reasoning might conclude that “evidence shows people above 65 years old experience X% more infections than young teenagers”, the counterfactual approach would predict “if all individuals were above 65 years old, there would be a Y% increase in infections”.

In this study, we apply causal BNs to a unique linked cross-sectional dataset from Kenya, Tanzania and Uganda which integrates an array of social, economic, behavioural and microbiological data to generate an evidence-based assemblage to explore how drivers of ABR in UTIs are interrelated^28^. Our systems-based approach consists of two objectives:

- Generate a causal BN of drivers of prevalence of MDR in UTIs from a combination of expert opinion and data.
- Use this causal BN to evaluate effects of simulated public health interventions.

## Methods

### Overview of Methodological Approach

**Figure 1:**
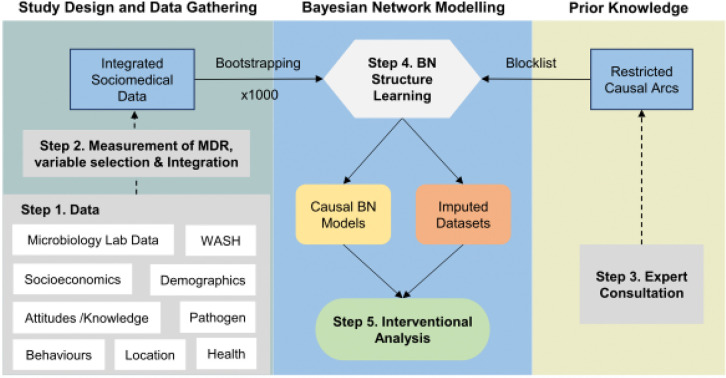
Flowchart of the methods.

Figure 1 provides an overview of the methodology.

Numbered steps described in text; further details can be found in the supplementary materials.

### Step 1. Data:

This study used data collected by the Holistic Approach to Unravel Antibacterial Resistance in East Africa (HATUA) Consortium, an interdisciplinary, three-country study on the drivers of ABR (full details described in the protocol^28^; sample selection and exclusions in Figure S1). Between February 2019 and September 2020, 6,827 adult outpatients (aged 18 years and older, or those 14–18 years and pregnant, who comprised 1% of the sample) were recruited from several healthcare facilities in three countries (Kenya: Makueni, Nairobi, Nanyuki; Tanzania: Kilimanjaro, Mbeya and Mwanza, Uganda: Mbarara, Nakapiripirit, and Nakasongola). Healthcare facilities were predominantly government-funded and included a variety of primary, secondary and tertiary healthcare facilities (see Table S1 for details and recruitment dates). Doctors or clinical officers identified patients with UTI-like symptoms for inclusion in the study. Less than 1% declined to participate, with no variation by location. Patients provided a mid-stream urine sample and answered a questionnaire on treatment-seeking, antibiotic use, health, knowledge and attitudes, demographics and household socioeconomic factors. Patient urine samples underwent microbiological culture, antibiotic susceptibility testing as reported elsewhere^4^ (see Table S2 for antibiotics tested). The majority were profiled genomically.

### Ethical approval

for this project was obtained from the University of St Andrews, UK (No. MD14548,10/09/19); National Institute for Medical Research, Tanzania (No. 2831, updated 26/07/19), CUHAS/BMC research ethics and review committee (No. CREC/266/2018, updated on 02/2019), Mbeya Medical Research and Ethics Committee (No. SZEC-2439/R. A/V.1/303030), Kilimanjaro Christian Medical College, Tanzania (No. 2293, updated 14/08/19). Uganda National Council for Science and Technology (number HS2406, 18/06/18); Makerere University, Uganda (number 514, 25/04/18); and Kenya Medical Research Institute (04/06/19, Scientific and Ethics Review Committee (SERU) number KEMRI/SERU/CMR/P00112/3865 V.1.2). For Uganda, administrative letters of support were obtained from the district health officers to allow the research to be conducted in the respective hospitals and health centres.

### Step 2. Measurement of MDR, variable selection and integration

The study outcome was phenotypically determined MDR UTI (binary classification) defined as urinary isolates resistant to at least one in three or more defined classes of antimicrobial agents, following the European Centre for Disease Prevention and Control (ECDC) guidelines^29^. We modified this to add nitrofurantoin and trimethoprim, two antibiotics routinely used for treating UTIs in this region. In addition, for species/genera not incorporated in the ECDC guidelines, i.e. *Salmonella* spp., *Shigella* spp., and *Streptococcus* spp., MDR was calculated as above, but considering resistance to a selected pool of tested antibiotics (Table S2). Isolates that showed intermediate resistance were considered resistant, following local clinical practice. In a sensitivity analysis, MDR was also measured genotypically (see page 5 of supplementary).

The selection of other variables was guided by the extant literature on drivers of ABR and MDR UTI^30–33^. We aimed to include variables with putative or established causal links to MDR. We included 27 variables measuring a range of factors: geographical and area-level (e.g., *site*), socioeconomic status (e.g., patient education), patient demographics (e.g., age, gender), health (e.g., chronic illness, recurrent UTIs), environmental/WASH conditions (e.g., toilet facilities), patient attitudes (e.g., UTI stigma), patient behaviours (e.g., recent medication use), as well as pathogen details (e.g., bacterial species) considered important for driving the outcome of MDR UTI. A description of the variables and their breakdown by gender/MDR status is provided in Supplementary Tables S3 and S4. After exclusions for missing linkages, we used a total of 2,007 samples containing linked questionnaire data, microbiological data from urine samples, socioeconomic and environmental data from patients’ households, and with geospatial data on location of clinics and households. There were only very small percentages of missing values in the final dataset (see Table S3). For sensitivity analysis, we used a subsample (n = 1,523) where MDR was defined genotypically.

Prior analysis of the HATUA data has shown that the variable *site* (which indicates which of the 9 districts where recruitment took place) acts as a strong confounder of the relationship between antibiotic use and MDR UTI^34^. *Site* may proxy for unmeasured area-level factors, potentially obscuring the causal relationships between MDR and other variables. In the interests of discovering causal relationships, for the main analysis we excluded location-based variables, including *hospital level*, because the distribution was dependant on site/country of recruitment. For example, in Kenya, due to COVID-19 restrictions, more recruitment happened in secondary and tertiary facilities than from primary care compared with other countries.

### Step 3. Expert consultation to inform BN network structure

We held consultation exercises with a multidisciplinary group of scientists to gather expert advice on causal relationships between the selected variables and inform subsequent BN structure learning. Experts identified variables that could not plausibly be causally related as per Pearl’s definition (either directly or through influence of other unmeasured factors), to inform a “block list” of variables (shown in Figure S2) that were restricted from being connected. This resulted in variables with similar features being grouped together and links restricted between entire groups. For instance, socioeconomic variables, such as education, cannot change demographic features like age and gender; therefore, no variables in the socioeconomic group were allowed to influence demographic variables.

### Step 4. BN Structure Learning:

To ensure the robustness of the constructed causal BN, we undertook a resampling process (‘bootstrapping’) using the original data. This generated 1,000 random resamples (‘bootstrapped datasets’), each containing 2,007 observations, 1,722 for females and 285 for males, as in the original data. For each bootstrapped dataset, we learned the BN structure (considering the block list from step 3) using the Structural Expectation Maximization algorithm, which imputes missing data during the structure learning process, leading to better network recovery and better imputation than imputing prior to structure learning^35^. We used the *structural.em* function of the R package *bnlearn*^36^ with parameters as follows: search was *hc* (hill climbing), scoring metric *bde* (BDe: Bayesian Dirichlet equivalent), parameter learning *mle* (MLE: Maximum Likelihood Estimation), maximum iterations 1,000, and maximum parents 3. Optimal *iss* values (impacts Bayesian prior) were calculated for each subset using our developed simulation method^37^ (see Supplementary Table S5, S6). An averaged network structure from 1,000 learned networks from the bootstrapped datasets was calculated using the averaged.network function^36^ with the default calculated threshold^36,38^ (0.483 for the overall population, 0.481 for females, and 0.498 for males). Complete, imputed datasets were extracted from the final iteration on each bootstrapped dataset. Networks (and imputed datasets) were calculated for the overall population as well as separately for males and females. All steps were repeated for subsamples of males, females and those with genomic data.

### Step 5. Interventional Analysis

We implemented the counterfactual approach for an interventional analysis of the causal effect of identified key factors on the prevalence of MDR UTI, calculating an intervention effect (IE) for each factor. The method was implemented in the Python *causalnex* library, utilizing the *InferenceEngine* class^39^. More details of this process are covered in supplementary material, and briefly described here.

**Figure 2:**
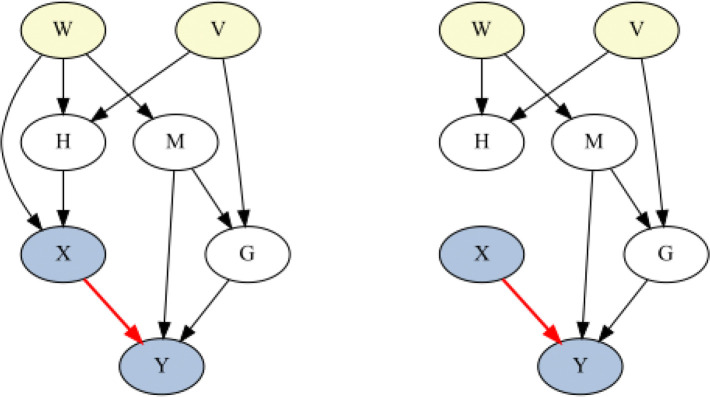
Hypothetical example showing how the IE is calculated with do-calculus.

To estimate the IE using BNs, confounders of the intervened and target variable are identified using either Pearl’s ‘back-door’ or ‘front-door’ criterion^22^. The intervention is calculated via ‘do-calculus’: all influences coming into the intervened variable are removed, to show that this variable is now being modified exogenously (‘graph surgery’), and the probability of the target variable calculated conditioned on the confounding variables for the fixed value of the intervened variable, resulting in a probability corresponding to the IE^40^. See example in Fig. 2.

A directed acyclic graph (DAG) (left) illustrating causal relationships between variables *X*, *Y*, *W*, *V*, *H*, *M* and *G*. The red arrow represents the causal effect of *X* on *Y*. To identify the IE, with X on Y highlighted in blue, one possible confounder set includes W and V, highlighted in yellow, based on Pearl’s back-door criterion. Do-calculus (right) calculates the probability of variable Y by hypothetically intervening on X, which removes incoming links to X via graph surgery and sets X to the intervened value, then determines the causal effect on Y while accounting for confounders W and V.

For each identified key causal factor, we computed the stratified (associative reasoning) and IE (counterfactual reasoning) probability of presenting with an MDR positive UTI (which, on a population level, corresponds to MDR prevalence) for each value of the factor for each learned network structure/imputed bootstrapped dataset pair; we calculated the difference of both from the overall prevalence of MDR in each imputed dataset. Because an IE can only be calculated on network structures where the intervened variable lies on the causal path of the target, only those learned network/bootstrapped dataset pairs where the key causal factor was in the causal path of MDR were analysed for both the stratified and IE analyses to ensure balanced comparison between associative and counterfactual reasoning. Results were aggregated across all learned network/bootstrapped dataset pairs to produce mean estimates of differences of the stratified and IE MDR prevalence compared to basal prevalence. This analysis was done for the overall dataset and also separately for males and females.

## Results

### Causal Bayesian networks for presenting with an MDR UTI

**Figure 3:**
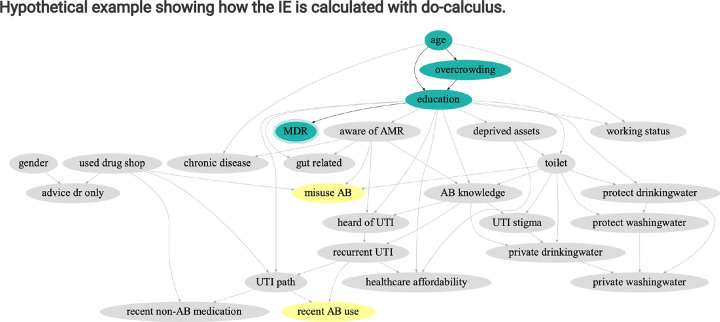
Average causal Bayesian network highlighting primary causal factors of MDR in the overall dataset.

The averaged causal BN generated for the overall sample (Fig. 3) reveals a complex network of interconnected socioeconomic, demographic, behavioural, clinical, and environmental factors. Age, overcrowding, and education emerged as primary causal factors of presenting with an MDR UTI, forging two main pathways: one from age to education to MDR, and another from age, through overcrowding, to education, and subsequently to MDR. Recent antibiotic use (within 6 months), rather than being a causal factor of presenting with an MDR UTI, was a result of recurrent UTI and patient treatment-seeking, with antibiotic misuse being directly influenced by the level of ABR awareness, the quality of toilet facilities, and the practice of purchasing antibiotics from drug shops. Neither antibiotic use nor misuse were direct or indirect causal factors of presenting with an MDR UTI. However, both shared all causal factors of MDR, upstream of additional factors including WASH and behavioural measures.

The green node with triple peripheries signifies the outcome, presenting with an MDR UTI. Green nodes represent primary factors appearing as direct or indirect causal factors of MDR in the average across all bootstrapped networks (age, overcrowded household, and education). Antibiotic use variables (recent AB use, misuse AB) are highlighted in yellow, influenced by other variables including those on causal pathways from the primary causal factors of MDR UTI.

**Figure 4:**
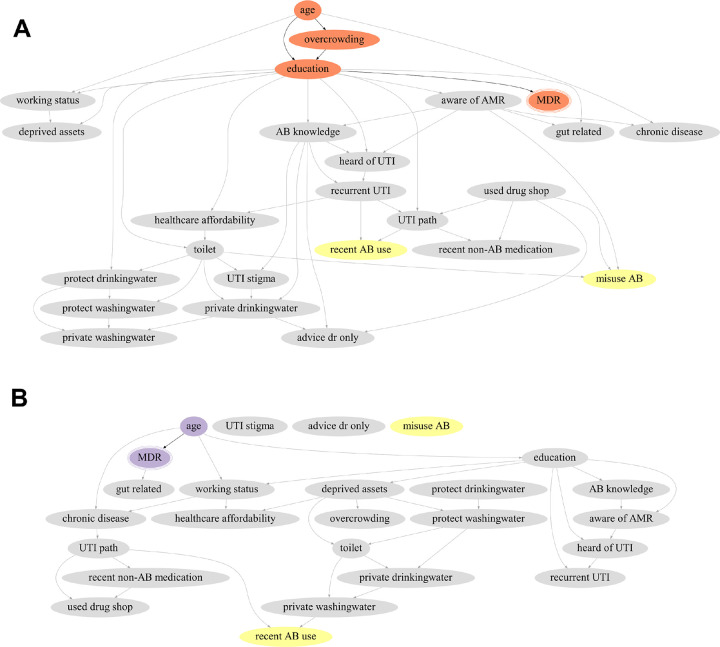
Average causal network highlighting direct causal factors of MDR in the female (panel A, n=1722) and male (panel B, n=285) datasets.

The averaged networks from the male- and female-only datasets revealed gender-specific patterns (Fig. 4). Among females (Panel A, comprising 86% of the full sample), the primary causal factors and their configuration of paths to presenting with an MDR UTI mirrored those identified in the overall population. Antibiotic use behaviours shared all of the causal factors, but did not directly influence MDR UTI. The averaged network for males (Panel B) was sparser with fewer connected nodes, attributed to a smaller sample size (285 observations compared to 1722 for females). Age emerged as the sole direct causal factor of presenting with an MDR UTI, aligning with findings from the overall and female-specific analyses. Again similar to the other analyses, recent antibiotic usage in males shared (the sole variable on) MDR’s causal pathway, upstream of treatment seeking process and WASH. Antibiotic misuse connected to no other variables; due to the low sample size, such isolation reflects interactions with this variable being of lower strength than those recovered, but also reinforces the observation that recent antibiotic usage and misuse did not contribute to the causal paths leading to MDR UTI in our patients.

**Figure 5:**
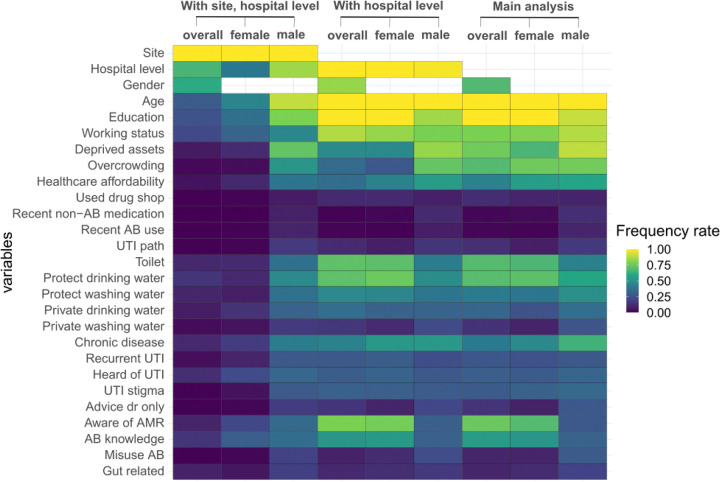
Frequency of variables appearing on MDR’s causal paths across different model configurations.

Outside of the primary causal factors seen in the averaged networks, additional nodes appeared along the causal paths of MDR in the 1000 bootstrapped analyses (Fig. 5). This highlighted additional influences of deprived assets, working status, access to protected drinking water, toilet facilities, and ABR awareness, which consistently appeared along the causal paths of MDR across population strata, suggesting they may also serve as effective intervention areas.

In panel A (females), the orange node with triple peripheries signifies the outcome, presenting with an MDR UTI. Orange nodes represent primary factors appearing as direct or indirect causal factors of MDR in the average across all bootstrapped networks (age, overcrowding, and education). Antibiotic use variables (recent use, misuse) are highlighted in yellow, influenced by other variables including those on causal pathways from the primary causal factors of MDR. In panel B (males), the purple node with triple peripheries signifies the outcome, presenting with an MDR UTI. The node in purple, age, represents the sole primary (and direct) causal factor of MDR identified in the average across all bootstrapped networks. Antibiotic use variables (recent use, misuse) are highlighted in yellow.

Figure 5 shows a heat map of the frequency across 1,000 bootstrapped datasets of each variable appearing on a causal path to MDR for nine model configurations (top labels). High consistency (similar coloured boxes across rows) was seen in the models with *hospital level* and the main analysis, with some differences in the male subset. *Site*’s presence on the causal path reduced the presence of other variables (except in the male subset to some extent).

### Sensitivity Analysis with Location Variables and Genomic data

**Figure 6:**
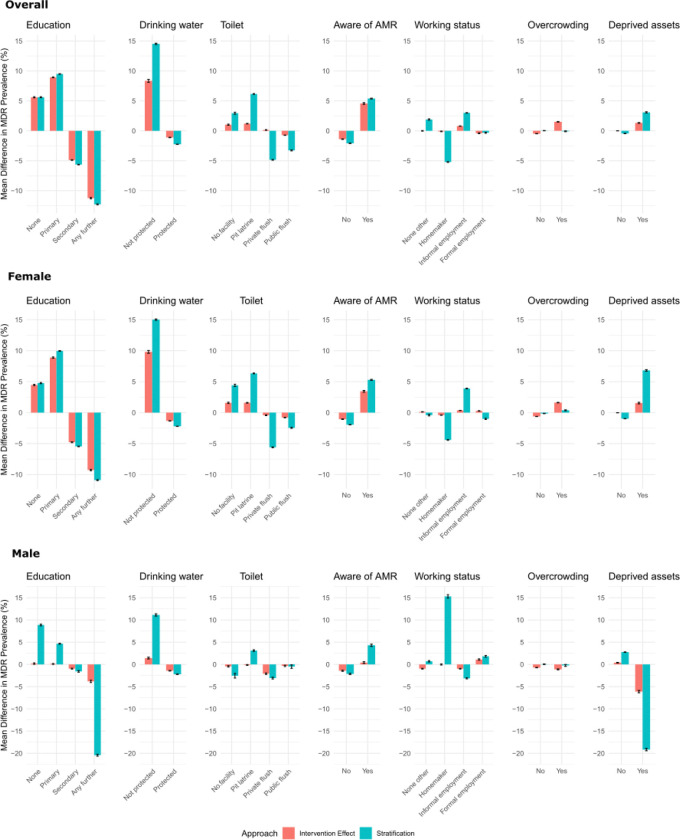
The impact of interventional analysis on MDR prevalence.

We also developed causal BNs including *site* and *hospital level* (Fig. 6, Figures S7-S12). *Site* was a strong confounder that connected to almost all nodes in the networks. In the overall and women’s subsets, it was also the only direct causal factor of MDR UTI identified. As above, antibiotic use and misuse were not on the causal path leading to MDR but emerged from a constellation of other factors also impacted by *site*. In males (Figure S9), *site* was not the only direct causal factor of MDR; there was an additional causal path from *site* to MDR mediated by *age*, and misuse of antibiotics was now connected in the network. The consistency of findings across various configurations demonstrates that the identified causal pathways are stable and reliable. We conducted similar analysis using genotypic characterisation of MDR on the whole sample and females only, and the causal structures were consistent (Supplementary Figures S14-S23).

### Interventional Analysis to calculate Intervention Effect (IE) on prevalence of MDR

Given Fig. 5 we chose to intervene on key causal drivers of: age, education, and household overcrowding (primary causal factors from averaged networks) plus drinking water, toilet facilities, AMR awareness, working status, and deprived assets (regularly present on causal path across all bootstrapped networks). IE of all factors potentially modifiable by public health interventions (e.g., minus age) are shown in Fig. 6. The process was repeated with the genomic data sample (Figure S24 & 25).

When we hypothetically intervened to ensure everyone had secondary or further education, the MDR prevalence decreased substantially by 5% (5% in females, 1% in males) and 11% (9% in females, 4% in males), respectively. Conversely, the MDR prevalence would be higher if all individuals had no education by 6% (4% in females, < 1% in males) or only primary education by 9% (9% in females, < 1% in males). This picture is consistent across gender-stratified models. Hypothetical intervention on working status had a minimal impact on MDR prevalence, with all effects of less than 1% in the overall population and gender-stratified-models. There was a mixed relationship between assets and MDR prevalence. In the overall and female populations, if all patients lived in households with deprived assets, it would increase MDR prevalence by 1%. Conversely, among males, universal deprived assets decreased MDR prevalence by 6%; however, the data underlying this inference were particularly sparse, with only 12.6% of men (36 of the 2,007 people in the sample) having deprived assets in the original data, leading to potential sampling artefacts. Eliminating household overcrowding led to a minimal (1%) decrease in MDR prevalence across all populations. Conversely, when everybody experienced household overcrowding, it increased MDR prevalence by 2% in the overall and female-only analyses. If no one had access to protected drinking water, MDR prevalence increased by 8% (10% in females; 1% in males). Conversely, MDR prevalence decreased by 1% in all populations if everyone had protected drinking water access. When everyone lacked toilet facilities or had pit latrines, MDR prevalence increased by around 1% and conversely, access to flush toilets was associated with a reduction (albeit minimal) in MDR prevalence across all populations. Somewhat counterintuitively, increasing awareness of ABR was associated with increased MDR prevalence (by 5% in the overall population, 3% in females). As age was identified as an important causal driver of presenting with an MDR UTI in the networks, we also investigated shifting the age distribution to be older (Figure S13), which shows that the MDR proportion would rise. These patterns are largely consistent when using the genotypic characterisation of MDR (Figures S24 and S25).

The IE largely align with the patterns observed from associative reasoning (stratification results), especially direction of effect. However, the magnitude of the effect varies, showing how counterfactual reasoning takes into account the network structure. For example, in the analysis on working status, the IE assuming of everyone having no other job/is a homemaker shows minimal difference in MDR prevalence, whereas in the stratified MDR prevalence in homemakers is lower than in the general population. These differences show the importance of including the web of interactions when estimating impact of potential interventions.

Mean percentage change in MDR prevalence due to intervention effect (IE) (red bars) compared to stratified analysis (blue bars) across potentially modifiable key causal drivers for the overall population (top) and disaggregated by gender (middle/bottom) calculated across bootstrapped datasets (error bars represent ± SE). Differences are shown from overall prevalence of 48% for overall, 46% women, 58% men. Factors are ordered left-to-right from larger to smaller IE impact. All variables are self-reported in the recruitment questionnaire by the patient. Education represents highest level of education obtained. Protected drinking/washing water indicates protected standpipe (not open to environmental contamination). Toilet represents the facilities the household uses on a day-to-day basis. Aware of AMR refers to whether individuals recognised the term antimicrobial resistance (AMR). Working status includes variations from none to formal employment (see labels). Overcrowding (a binary) is measured by dividing the number of household members by sleeping rooms, with 3 or more people per room classed as ‘overcrowded’. Deprived assets is measured by not owning more than one of the following assets: television, radio, computer, fishing boat, telephone, refrigerator, or bed), following HATUA multidimensional poverty definition used elsewhere^38^.

## Discussion

This study used data on UTI patients in East Africa to investigate causal drivers of presenting with an MDR UTI. We established a causal BN of MDR drivers informed by expert opinion, then evaluated effects of hypothetical interventions on MDR prevalence while taking account of network structure connecting behavioural, demographic, socioeconomic and environmental variables. We identified age, education, and household overcrowding as important primary causal factors. The network also highlighted that antibiotic use and misuse were not important causal factors of presenting with an MDR UTI at the individual level; they were instead influenced by other behavioural and community level factors and shared upstream drivers with MDR prevalence. The interventional analysis suggested that increasing the proportion of people with secondary level education, enhancing access to protected drinking water and toilet facilities, and decreasing household overcrowding could help to reduce prevalence of presenting with an MDR UTI.

The causal Bayesian networks used here provide a quantitative methodological tool to investigate ABR as an interconnected system of human, animal, and environmental health domains, aligning with ‘One Health’, ‘assemblage’ and ecosystem-based approaches^5,41^. This approach can do justice to the complex web of interrelationships and provide a more nuanced framework to develop interventions. Through this, for example, we can see that historically prioritised intervention points like antibiotic use sit within a wider web of causative factors. While antibiotic use and misuse have links from demographic, socioeconomic, and environmental factors at the community level, they do not link directly to presenting with an MDR infection. Rather, they share upstream causative factors with MDR prevalence. This suggests that presenting with an MDR infection is more impacted by factors influencing clinical vulnerability and bacterial transmission. Antibiotic use and misuse – known biologically to impact selection for MDR – is impacted by both those factors affecting presenting with an MDR UTI plus wider sociodemographic features. This can help us understand why interventions promoting “rational” use of antibiotics^42^ through ABR stewardship and knowledge-based campaigns might fail to reduce ABR prevalence: already existing ABR bacteria continue to be transmitted and infect in the short term, washing out any impact of reduced selective pressures which only appear in long term analyses^43^. At the same time, if transmission of infections overall continues to be high, higher pathogen population size leads to faster response to selection. The analysis also suggests that causal networks could be highly context-dependant, and thus, interventions should be carefully tailored to that context. When location (e.g. *site*) variables are included, the causal dynamics shift, suggesting important unmeasured causal factors that could be incorporated in interventions once known. Our models also reveal distinct causal networks for men and women, suggesting the potential for gender-specific and other subgroup interventions.

Our approach also highlights the potential efficacy of intervening on upstream social and public health dimensions to tackle MDR/ABR prevalence in UTIs. In particular, hypothetical interventions enhancing access to protected drinking water and flush toilet facilities significantly reduced the MDR prevalence across all populations, aligning with reviews and modelling studies proposing a focus on existing public health issues like WASH in LMIC settings^10,44^. Beyond infection prevention and control, our study also points to broader social determinants of health, e.g., education access. This is a multifaceted development intervention which impacts ABR in complex ways through individual and community dynamics: population processes, gender equity, health, socio-economic resources, labour force participation, migration, and more^45^. Likewise, household overcrowding also appears as an important causal factor in the network, consistent with other studies that have found associations between overcrowding and ABR^46,47^. UTI infections are predominantly due to gut-related Gram-negative bacteria. Overcrowding, through enhanced contacts and increasing the risk of diarrhoeal disease, might increase chances of transmitting UTIs. A somewhat counterintuitive finding was that increased ABR awareness was associated with higher MDR prevalence. This could be explained by reverse causality, given that it was familiarity with the term not deeper understanding which was recorded, such that patients with MDR infections have become aware of ABR through healthcare contacts. Relatedly, we also observe higher ABR awareness among patients with a history of antibiotic use compared to those without (0.31 vs. 0.24 in the overall population, 0.29 vs. 0.24 in females, and 0.38 vs. 0.24 in males), which could be jointly determined by contexts of higher infection and greater need for antibiotics. Regardless, there does not seem to be a straightforward relationship in this data between knowledge of ABR and its prevalence.

This study also highlights some philosophical and practical issues in studying causation for real-world ‘wicked’ problems like ABR, when experimental conditions are impractical and unethical, and when webs of causation are very complex. The underlying logic of causal inference applied here draws on Pearl’s work^22^ on causal reasoning, and in doing so we represent causal relations mathematically using DAGs and causal BNs. The advantage of this approach is we do not have to rely on restrictive distributional assumptions, and we can allow variables/nodes to have multiple complex connections. We can learn relationships from the data while being able to incorporate domain expertise to partially guide this structure learning, making directionalities more plausible (important as we were using cross-sectional data). This demonstrates the possibilities of observational data analysis for inference of effects of hypothetical interventions, by combining data-driven approaches, Bayesian causal logic and expert input. The causal modelling and intervention methodology are adaptable to a wide range of research scenarios on ABR across various geographical and healthcare settings, which would benefit from understanding ABR as a system. The challenge of applying this approach is that Pearl argued one can identify causality with complete information; however, complete information is a theoretical ideal which no sampled dataset can achieve. Nevertheless, the methodology can still provide useful inferences and insights in the ‘real world’. When using a sampled dataset to inform structure as in this study, we must retain awareness that we may not have measured every important factor adequately and our sample may not be large enough or representative enough of the population we are trying to generalise to. When using experts to define potential causal paths, the network will reflect the assumptions given, including allowing connections through unmeasured variables. For example, the male subset in this study was considerably smaller and the causal links inferred sparser, strongly suggesting many missed interactions compared to the female or overall dataset. As another example, the sensitivity analysis including site and hospital level, while supporting the robustness of the overall structure and causal drivers identified in the main analysis, also pointed to existence of unmeasured, more direct drivers associated especially with site. Thus, we must balance awareness of the limitations of the sampled dataset used (and thus potential missed factors) with the powerful ability to generate complex webs of interactions explaining relationships within the data and to see effects of hypothetical interventions.

Some limitations of our study relate to the HATUA dataset which include: (a) sampling exclusively from patients attending healthcare facilities, which may not represent the broader community; (b) using UTI as a prism for ABR more generally, necessitating replication with other types of infections to generalize findings; (c) the sampling in Kenya was more urban-centric compared to the other locations, and included fewer primary care facilities; and (d) different attrition and missing data patterns across the sites. Despite measuring a plethora of individual, household and community variables, analyses are likely still subject to unmeasured confounding. In particular, we measured geographic variability using a categorical indicator for *site* which may proxy for other infrastructural factors that vary by location. These limitations speak to the difficulties of identifying causal factors but emphasise the need to address them with better designed studies. For example, future analyses would benefit from direct measurement of geographical and infrastructural factors to unpick place-based processes.

This study used a network-based modelling approach to identify causative factors of MDR in UTIs in a real-world African community setting. It demonstrated the power of causal BNs and interventional analysis to investigate and design solutions to complex problems like ABR. The modelling approach revealed networks of causal drivers that are gender-specific, highlighted context-dependant nature of some interactions, and revealed hypothetical interventions with impact on MDR prevalence. The most effective intervention points rested on already established infrastructural public health and social initiatives such as WASH and socioeconomic status.

## Data Availability

Analysis code is available on github: [insert url] The data that support the findings of this study is available according to data sharing policy of the partners in the three participating countries, which restricts access due to ethical issues. The data forms part of a larger linked dataset, with ongoing analysis. To request access, please contact the Principal Investigator of the HATUA Consortium Professor Matthew Holden (mtgh@st-andrews.ac.uk) or the corresponding author (katherine.keenan@st-andrews.ac.uk). At the time of publication, further reuse of the data for analysis would require collaboration in the ongoing work of the HATUA Consortium.

## Consortium Authorship

This is the full list of HATUA Consortium members, in alphabetical order:

David Aanensen^8^; Annette Aduda^5^; Benon Asiimwe^3^; Alison Elliott^10,11^; Kathryn J. Fredricks^1^; Stephen H. Gillespie^1^; Dominique L. Green^1^; Matthew T. G. Holden^1^; Catherine Kansiime^3^; Katherine Keenan^1^; Mike Kesby^1^; Gibson Kibiki ^7^; John Kiiru^5^;Andy G Lynch^1^; John Maina^5^;Blandina T. Mmbaga^6^;Stephen E. Mshana^2^;Martha F. Mushi^2^; Joseph R. Mwanga^2^; Stella Neema^3^; Wilber Sabiiti^1^;Alison Sandeman^1^; Derek J. Sloan^1^;V Anne Smith^1^;John Stelling ^9^

^1^ University of St Andrews, St Andrews, UK

^2^ Catholic University of Health and Allied Sciences, Mwanza, Tanzania.

^3^ Makerere University, Kampala, Uganda.

^4^ Mbarara University of Science and Technology, Mbarara, Uganda.

^5^ Kenya Medical Research Institute, Nairobi, Kenya

^6^ Kilimanjaro Clinical Research Institute, Kilimanjaro Christian Medical Centre, Moshi, Tanzania; Kilimanjaro Christian Medical University College, Moshi Tanzania

^7^ Africa Excellence Research Fund, London, UK

^8^ Oxford Big Data Institute, Oxford, UK.

^9^Brigham and Women's Hospital, Boston, United States.

^10^ London School of Hygiene and Tropical Medicine, UK

^11^ MRC/Uganda Virus Research Institute (UVRI) , Uganda
